# Bee Body Size and Foraging Behavior Predict the Pollination Role of Bees in a Buzz‐Pollinated Plant Community

**DOI:** 10.1002/ece3.72150

**Published:** 2025-09-08

**Authors:** José Neiva Mesquita‐Neto, Clemens Schlindwein

**Affiliations:** ^1^ Laboratorio Ecología de Abejas Nativas, Departamento de Biología y Química, Facultad de Ciencias Básicas Universidad Católica del Maule Talca Chile; ^2^ Departamento de Botânica, Grupo Plebeia—Ecologia de Abelhas e da Polinização Universidade Federal de Minas Gerais Minas Gerais Brazil

**Keywords:** buzz‐pollination, Cerrado, effective pollinators, Fabaceae, Melastomataceae, Ochnaceae, pollen thieves, poricidal anthers

## Abstract

Buzz pollination has been studied for over a century, yet the drivers shaping plant–pollinator interactions at the community level remain poorly understood. Although previous research has identified key functional traits associated with sonication‐foraging behavior and taxonomic relationships among floral visitors, the specific bee traits that promote effective pollination are still unclear. In this study, we investigated which morphological and behavioral characteristics of flower‐visiting bees predict pollination success within a plant community containing multiple buzz‐pollinated species. Focusing on eight co‐occurring, co‐flowering plant species with poricidal anthers, we measured stigma contact (as a proxy for pollination efficiency), visitation rates, flower and whole plant handling time, intertegular span (body size), and pollen extraction mode across different bee species. Our results showed that both morphological and behavioral traits can predict pollination success. Specifically, stigma contact was positively associated with larger body size, suggesting that larger bees are more likely to touch the stigma during visits. In contrast, longer flower and plant handling times were linked to a low likelihood of stigma contacts—traits more commonly observed in smaller bees. Bees that used floral sonication visited more flowers and plants per unit time and contacted stigmas approximately four times more frequently than non‐sonicating bees. However, detailed aspects of sonication behavior—such as the number and duration of buzzes—were not associated with pollination efficiency. These findings suggest that while bees visiting buzz‐pollinated flowers may share similar pollen extraction mechanisms, variation in physical and behavioral traits plays a crucial role in shaping pollination dynamics.

## Graphical abstract

We investigated how bee traits influence pollination success in a buzz‐pollinated plant community. Larger body size and floral sonication strongly predicted stigma contact, a proxy for pollination efficiency, while longer handling times were associated with reduced effectiveness. Our findings highlight the importance of both morphological and behavioral variation in shaping pollination outcomes.

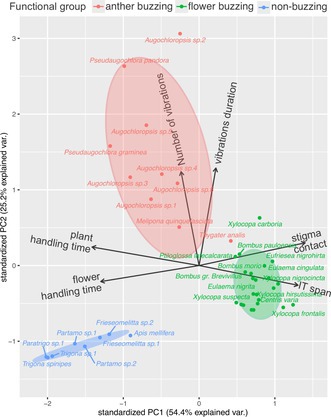

## 1 Introduction

More than 20,000 plant species across multiple families possess poricidal anthers, which release pollen exclusively through small apical pores (Buchmann 1983; Vallejo‐Marín et al. 2010; Russell et al. 2017). The most effective mechanism for pollen release is floral vibration, primarily performed by bees—a phenomenon known as buzz pollination or sonication (Buchmann and Cane 1989; De Luca and Vallejo‐Marín 2013; Vallejo‐Marín 2019). During flower visits, these bees activate their thoracic muscles to vibrate the anthers, extracting large quantities of pollen (Michener 1962; Buchmann and Hurley 1978; Vogel 1978; Renner 1983; Snow and Roubik 1987; Harder and Barclay 1994; De Luca and Vallejo‐Marín 2013; Burkart et al. 2014; Solís‐Montero and Vallejo‐Marín 2017). However, bees do not vibrate all flowers equally. Differences in sonication are likely due to variation in floral morphology and biomechanics, as well as differences in pollen availability and rewards (Kawai and Kudo 2009; Burkart et al. 2011, 2014; Switzer and Combes 2016; Switzer et al. 2019; Arroyo‐Correa et al. 2019; Vallejo‐Marín 2022; Russell et al. 2016).

Buzz pollination has been studied for over a century (Lindman 1902; Teppner 2017; Portman et al. 2019), yet key questions remain—particularly regarding the relationship between pollen extraction and pollination efficiency. Buzz‐pollinated plants are typically nectarless and rely almost exclusively on pollen as a pollinator reward (Harter et al. 2002; Vallejo‐Marín et al. 2010; De Luca and Vallejo‐Marín 2013). This creates a potential conflict: pollen is both a reward for pollinators and the medium for plant reproduction (Müller 1881, 1883; Klinkhamer and de Jong 1993; Westerkamp 1997; Barrett 2010). Pollen used as food is typically unavailable for fertilization, and vice versa (Endress 1996), which may explain why efficient pollen collection by bees does not always translate into effective pollination (Mayberry et al. 2024).

Additionally, the extent of variation in bee traits related to buzz pollination is still poorly understood, as the sonication behavior of many species remains uncharacterized (De Luca and Vallejo‐Marín 2013; Vallejo‐Marin and Russell 2023). Approximately 58% of the known bee species—around 11,600 species across 74 genera—are estimated to remove pollen by flower sonication (Cardinal et al. 2018), particularly within the tribes Centridini, Euglossini, Xylocopini, Bombini (Apidae), Augochlorini (Halictidae), and Caupolicanini (Colletidae) (Michener 1962; Vogel 1978; Buchmann 1983). Many of these taxa are found in the Neotropical region, which also hosts high taxonomic and morphological diversity of buzz‐pollinated plants. Yet, most buzz pollination studies have focused on a small number of native or cultivated plant families (Renner 1983; Arceo‐Gómez et al. 2012; Burkart et al. 2014; Solís‐Montero et al. 2015; Solís‐Montero and Vallejo‐Marín 2017; Staines et al. 2017; Mesquita‐Neto et al. 2021) or on the biomechanical properties of buzzing in a few bee species (Arceo‐Gómez et al. 2011; Burkart et al. 2011; Vallejo‐Marin and Russell 2023; Vallejo‐Marin et al. 2024). In contrast, broader patterns—such as pollinator specificity, pollination efficiency, and regional foraging behavior—remain understudied (Harter et al. 2002; Mesquita‐Neto et al. 2018; González‐Vanegas et al. 2021; Vallejo‐Marin and Russell 2023; Russell et al. 2024).

Research on buzz‐pollinated communities suggests that plant‐bee interactions are non‐random and structured by subgroups of interacting species (Mesquita‐Neto et al. 2018). While plants with poricidal anthers share a specialized pollen release mechanism, their floral visitors vary. Prior studies have addressed the foraging behavior and taxonomic relationships of floral visitors (Mesquita‐Neto et al. 2018), but the specific bee traits that drive pollination success remain unclear. Thus, our aim was to identify the physical and behavioral traits of flower‐visiting bees that predict pollination success in a buzz‐pollination plant community.

Using the same plant‐pollinator community studied by Mesquita‐Neto et al. (2018) in a natural area of Brazilian Cerrado, here we include key bee traits that can serve as proxies for pollination efficiency (stigmatic contact), including body size and foraging behavior, specifically the biophysical properties of sonication pulses (number and duration of pulses) and the time spent handling a flower and foraging on an individual plant during a visiting bout. Among the morphological traits, body size seems to be particularly relevant as it has been linked to pollination efficiency in several studies (Garibaldi et al. 2015; Jauker et al. 2016; Földesi et al. 2020; Mesquita‐Neto et al. 2021). Larger pollinators tend to deposit more pollen on stigmas (Földesi et al. 2020). Furthermore, the intricate floral structure of buzz‐pollinated flowers limits the range of effective pollinators, requiring precise pollen deposition on the body of a visiting insect for successful transfer to another flower (De Luca and Vallejo‐Marín 2013). This suggests that pollination success is influenced not only by the presence of sonication behavior (as demonstrated by Mesquita‐Neto et al. 2018), but also by the size of the visitor relative to the spatial arrangement of the reproductive organs of the flower (Mesquita‐Neto et al. 2021; Rego et al. 2024). Therefore, we hypothesize that bee body size is an important functional trait in buzz pollination and that larger bees are more effective pollinators than smaller bees (Hypothesis 1).

Foraging time per flower (or flower handling time) may also influence pollination success, as longer visits could increase the likelihood of stigma contact (Woodcock et al. 2013). However, there is evidence that longer visits do not necessarily increase pollen deposition (Thomson and Goodell 2001; Mesquita‐Neto et al. 2021). Despite limited evidence supporting the importance of this trait (Greenop et al. 2023), we hypothesize that bees spending more time handling a flower will be more effective pollinators of plants with poricidal anthers than those that visit flowers for shorter durations (Hypothesis 2).

## 2 Material and Methods

### 2.1 Study Site and Plant Species

Fieldwork was conducted from September to December in 2014 and 2015 in the Rio Preto State Park (Parque Estadual do Rio Preto), located in the municipality of São Gonçalo do Rio Preto, Minas Gerais, Brazil. The region has a tropical climate (Aw, Köppen classification; Peel et al. 2007), with a dry season during the cooler months, a rainy season during the warmer months, and average monthly temperatures ranging from 18°C to 30°C. The vegetation of the park is characteristic of the Cerrado (Brazilian savannah).

We included in this study all co‐flowering plant species with poricidal anthers that had at least 10 individuals within 500 m of a central point (18°05′28.3″S, 43°20′29.2″W). Eight species from three botanical families met these criteria: (1) Fabaceae: *Chamaecrista debilis* (Vogel) H.S. Irwin & Barneby, *Chamaecrista ramosa* (Vogel) H.S. Irwin & Barneby; (2) Melastomataceae: *Comolia stenodon* (Naudin) Triana, *Lavoisiera imbricata* DC., *Macairea radula* (Bonpl.) DC., *Miconia albicans* (Sw.) Steud., *Miconia tococa* (Desr.) Michelang; (3) Ochnaceae: *Ouratea floribunda* Engl.

### 2.2 Bee Morphological and Behavioral Traits and Pollination Efficiency

We sampled floral visitors from 10 individuals of each of eight plant species across the full daily activity period, from 4:30 to 18:00, on 56 sunny days in 2014 and 2015. A collector used an entomological net to capture all floral visitors during 15‐min observation sessions at each individual plant. After each session, the collector moved to an individual of a different plant species and continued this cycle until 18:00.

For each flower visit, we recorded whether the bee touched the stigma, the time elapsed between landing and departing from a flower (flower handling time), and the total time spent visiting flowers on the same individual plant during a single bout (plant handling time). In addition, we measured the number and duration of buzzing pulses with a hand‐held chronometer when the visitor engaged in buzzing behavior. In the laboratory, we identified bees to the lowest possible taxonomic level and measured their intertegular spans using a digital caliper as a proxy for body size (Cane 1987).

The classification of foraging behavior was based on the definitions provided by Mesquita‐Neto et al. (2018), without modifications. This study defined three functional groups of visiting bees based on their pollen collection behavior: flower‐buzzing bees (bees that sonicate all anthers of a flower in a fixed position), anther‐buzzing bees (bees that sonicate individual anthers or a set of a few anthers of a flower at once, changing their position in the flower) and non‐buzzing bees (bees that collect pollen without sonicating the anthers).

For plants that rely on insect pollination, stigma contact is a prerequisite for pollination success (Russo et al. 2017; Artz and Nault 2011; Greenop et al. 2023).

We then used the proportion of visits with stigma contact for each visiting bee species as a proxy for pollination efficiency.

### 2.3 Statistical Analysis

We first used a Principal Component Analysis (PCA) to reduce the dimensionality of the dataset while preserving the physical and behavioral traits of flower‐visiting bees important for pollination success (stigma contact). We quantified “stigma contact” as the proportion of times a given bee species made contact with the stigma divided by the total number of observed visits. In the PCA plot, we color‐coded the flower‐visiting bee species according to their assigned functional group (flower‐buzzing, anther‐buzzing or non‐buzzing), enabling visual comparison of trait variation across groups. We then assessed the normality of the response variable (stigma contact) using QQ plots and the Shapiro–Wilk test. Since it did not follow a normal distribution, we applied statistical methods designed for non‐normal data.

To assess the relationships between morphological and behavioral traits of flower‐visiting bees (flower handling time, plant handling time, intertegular span, number and duration of buzzing pulses), we calculated Spearman correlation coefficients (rs) for each pair of variables using the cor() function from the corrr package. The significance of each correlation was tested using two‐tailed *p*‐values with the cor_pmat() function. In this test, a significant correlation between a given trait of flower‐visiting bees would indicate that this variable influences stigma contact (our proxy of pollination efficiency). A Spearman's coefficient (rs) equal to or less than 0.3 is interpreted as a weak effect size; rs = 0.6, moderate effect size; and rs = 0.6 onwards, strong effect size.

We used a generalized linear mixed model (GLMM, ordered beta regression distribution, link = “logit”) to determine whether three functional groups of visiting bees (predictor variables) differed from each other in the proportion of visits with floral stigma contact (function “glmmTMB”, “glmmTMB” package; Brooks et al. 2023). We included bee taxon as a random effect. All statistical analyses were performed using R software version 4.1.2 (R Core Team 2022).

## 3 Results

**FIGURE 1:**
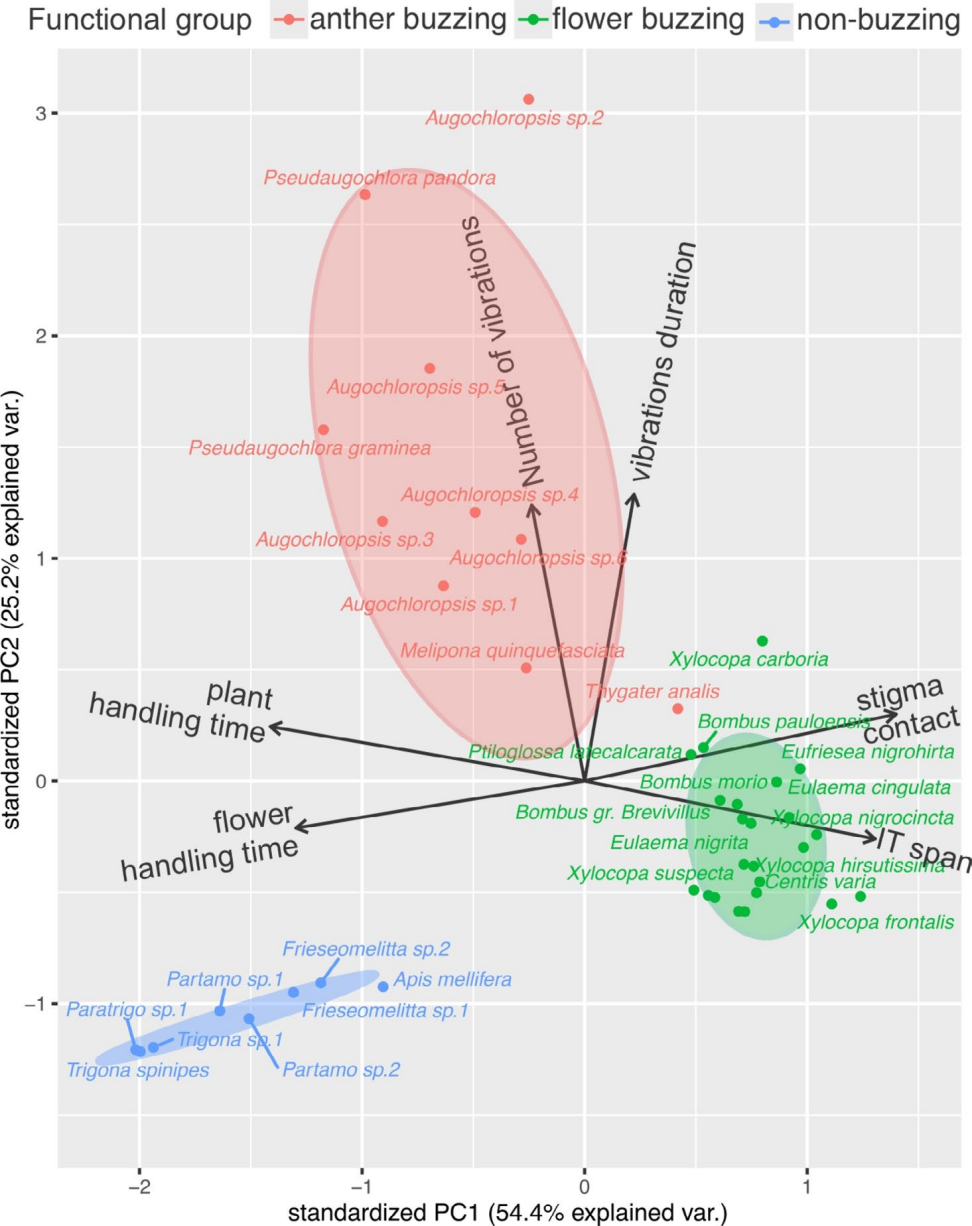
Principal component analysis (PCA) of behavioral and morphological traits of bees visiting buzz‐pollinated plants in Rio Preto State Park, Minas Gerais, Brazil, represented along two principal components (PC1 and PC2). The analysis includes a proxy for pollination efficiency (stigma contact) and bee traits from the three functional groups of visiting bees (flower‐buzzing, anther‐buzzing, non‐buzzing), including body size (intertegular span), number and duration of buzzing pulses, and time spent handling flowers and individual plants. Ellipses represent the 95% confidence intervals for each bee functional group. PC1 (54.4%) and PC2 (25.2%) together explain 79.6% of the total variance in the data.

During 720 h of sampling effort, we collected 442 bee individuals from 55 species and 11 tribes on the flowers of eight plant species with poricidal anthers (Table S1). Our PCA analysis revealed that the three functional groups of flower‐visiting bees differed in body size (intertegular span) and foraging behavior (Figure 1). Flower‐buzzing bees were larger in body size and were the group most associated with higher stigma contact. Longer flower and plant handling times were associated with small, non‐buzzing bees. In contrast, anther‐buzzing bees were more associated with longer vibration pulses.

### 3.1 Flower and Plant Handling‐Times

**FIGURE 2:**
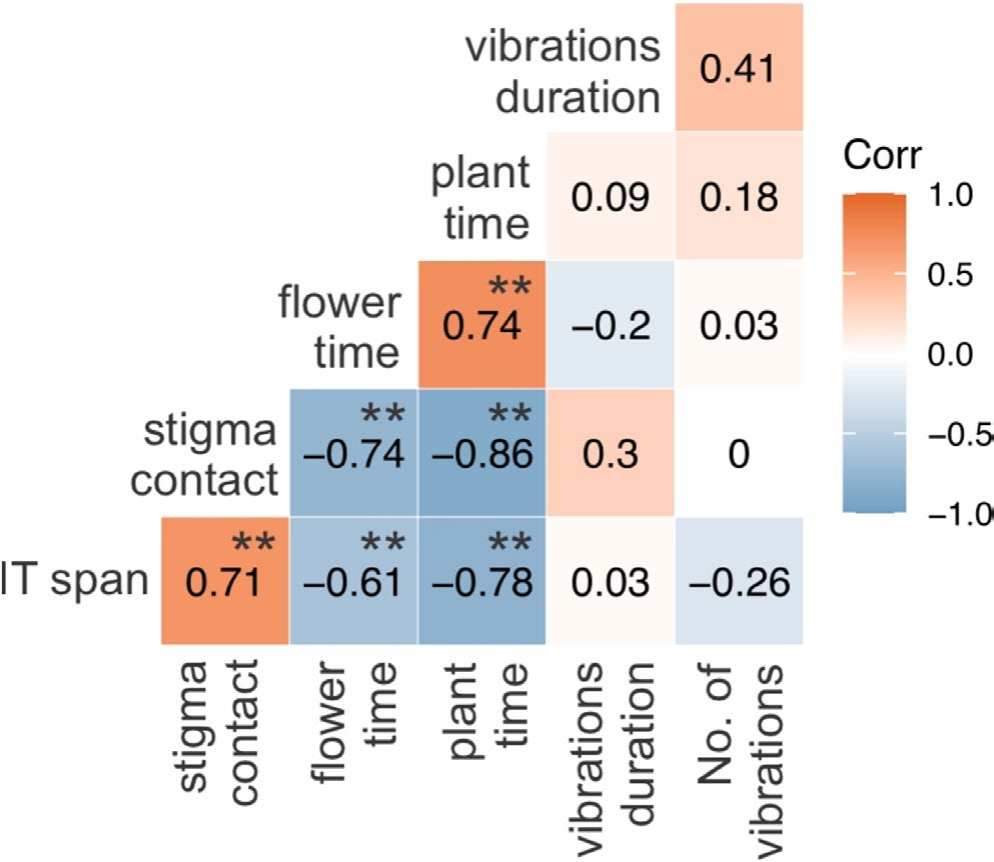
Correlation matrix between bee traits—body size (intertegular span), number and duration of buzzing pulses, and time spent handling flowers and individual plants—and their pollination efficiency (stigma contact) during visits to a community of buzz‐pollinated plants in Rio Preto State Park, Minas Gerais, Brazil. Correlation values range from −1 to 1, as indicated by the color bar, with positive values (orange) representing positive correlations and negative values (blue) indicating negative correlations, based on Spearman's correlation. Color intensity reflects the strength of the correlation, and the values within cells represent the exact correlation coefficients. Asterisks (**) denote statistically significant correlations (*p* ≤ 0.05).

There was a strong negative correlation between bee size (intertegular span) and flower handling time (rs = −0.61, *p* ≤ 0.001; Figure 2). Thus, smaller bees tended to have longer flower handling times and vice versa. The average flower handling time of flower‐buzzing bees was 4.1 s (±5.9 s), 7.5 times shorter than that of non‐buzzing bees (30.6 ± 25.7 s) and 2.9 times shorter than that of anther‐buzzing bees (12.0 ± 6.1 s; Figure 2).

There was a strong negative correlation between intertegular span and plant‐handling time (rs = −0.78, *p* ≤ 0.001; Figure 2). Smaller bees spent more time per individual plant than larger bees. Non‐buzzing bees stayed 1.5 times longer visiting flowers of the same plant (60.0 ± 0.1 s) than anther buzzing bees (40.0 ± 16.8 s) and 4.5 times longer than flower buzzing bees (13.3 ± 11.6 s).

### 3.2 Stigma Contact

**FIGURE 3:**
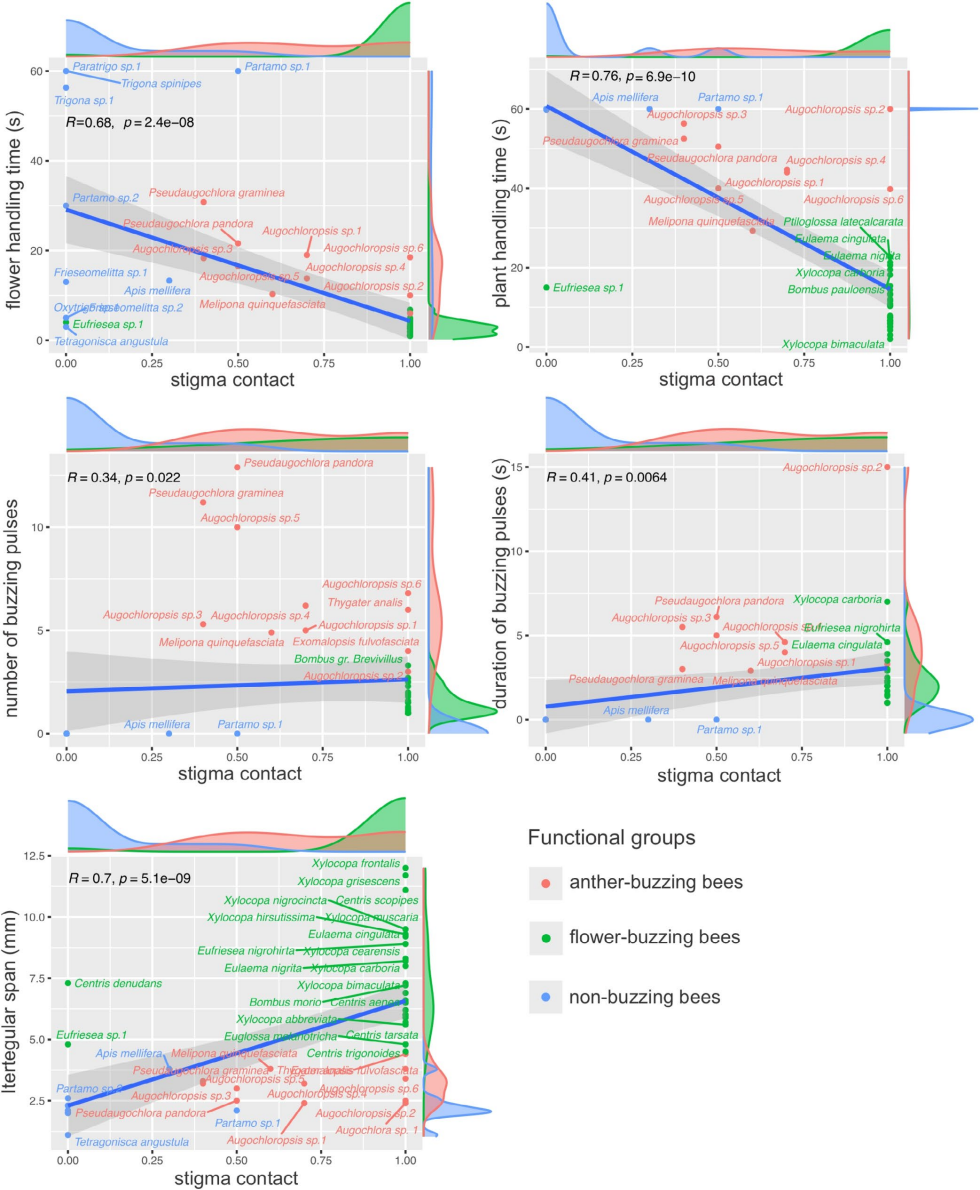
Pairwise Spearman rank correlations between morphological (intertegular span) and behavioral traits (number and duration of buzzing pulses, and time spent handling flowers and individual plants) of the three functional groups of bees (flower buzzing, anther buzzing, and non‐buzzing bees) and their pollination efficiency (stigma contact) in a community of plants with poricidal anthers at Rio Preto State Park, Minas Gerais, Brazil. Each point represents the mean value from visits by different individuals of a given bee species. Solid blue lines indicate the correlation trend, and the shaded areas represent 95% confidence intervals.

We found a strong negative correlation between stigma contact and flower and plant handling times (Figure 3). Thus, longer flower handling times are associated with lower chances of stigma contact. Stigma contact was positively associated only with intertegular span, indicating that larger bees more frequently touched the stigma during visits (Figure 3). Plant and flower handling times were also strongly positively related. Intertegular span was also strongly negatively associated with flower and plant handling times (Figure 3). The number and duration of flower vibrations were not significantly associated with any of the variables measured here (Figure 3).

**FIGURE 4:**
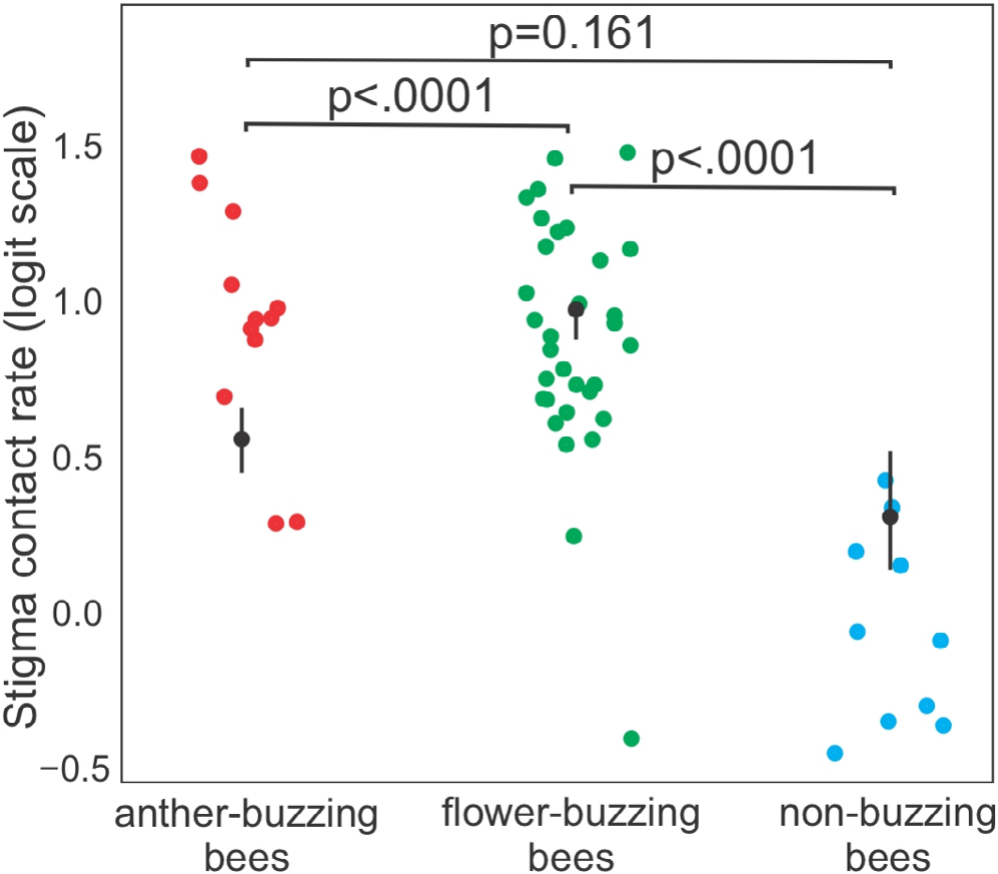
Estimated marginal means (with 95% confidence intervals) from the GLMM model assessing the effect of the three functional groups of bees (flower buzzing, anther buzzing, and non‐buzzing bees) on pollination efficiency (measured as stigma contact) in plants with poricidal flowers at Rio Preto State Park, Minas Gerais, Brazil. Each point represents the average proportion of visits with stigma contact for a given bee species. Note that values are shown on the logit scale, as estimated by the model with a beta distribution and logit link function. This can result in values falling outside the 0 to 1 range, even though raw proportions are constrained between 0 and 1. The proportion of visits with stigma contact differed significantly among functional groups (glmmTMB: *χ* ^2^ = 18.49, df = 2, *p* < 0.001).

The proportion of visits with stigma contact varied among the functional groups of visiting bees (glmmTMB: *χ* ^2^ = 18.49, df = 2, *p* < 0.001). Non‐sonicating bees touched the stigma in 8.7% (±17.8) of flower visits, while anther buzzing bees and flower buzzing bees touched the stigma in 72.8% (±25.5) and 93.5% (±24.5) of flower visits, respectively. Flower‐buzzing bees were substantially more likely to contact the stigma than non‐buzzing bees (flower buzzing/non‐buzzing: Odds Ratio = 4780.9 ± 9430, z‐ratio = 4.29, *p* = 0.0001) and also more likely than anther buzzing bees (anther buzzing/flower buzzing: OR = 0.001 ± 0.002, z‐ratio = −3.865, *p* = 0.0003; Figure 4). No difference was found among anther buzzing bees and non‐buzzing bees (anther buzzing/non‐buzzing: OR = 5.53 ± 5.17, z‐ratio = 1.8, *p* = 0.1610; Figure 4).

## 4 Discussion

Although sonication behavior is crucial for pollination efficiency in buzz‐pollinated plants (Stubbs and Drummond 2001; Cooley and Vallejo‐Marín 2021; Javorek et al. 2002; Cortés‐Rivas et al. 2022), our results showed that other characteristics of sonication behavior, specifically the number and duration of pulses, were unexpectedly unrelated to pollination efficiency. Even more unexpectedly, longer flower and plant handling times negatively reduced the probability of stigma contact, exactly the opposite trend to what our Hypothesis 2 predicted.

### 4.1 No Improvement in Pollination due to Number and Duration of Sonication Pulses

Besides the known close relationship between the presence of poricidal anther dehiscence and the presence of buzzing behavior, with the vibrational characteristics of the pulses directly affecting pollen extraction (Harder and Barclay 1994; De Luca and Vallejo‐Marín 2013), it is surprising that the number and duration of pulses did not lead to more stigmatic contact. This is particularly unexpected because the amount of pollen released in many plant species is significantly influenced by the duration of buzzing (De Luca and Vallejo‐Marín 2013; Tayal and Kariyat 2021), with bees able to fine‐tune the mechanical properties of their floral vibrations to optimize energy use and pollen collection (Vallejo‐Marín 2022). This relationship explains why bees increase buzz duration and the number of buzzes when they perceive that flowers still have pollen available, for example (Buchmann and Cane 1989; Harder 1990; Shelly et al. 2000; Nunes‐Silva et al. 2013; Burkart et al. 2014). However, our results showed that the duration of the vibrational pulses did not lead to better pollination efficiency (more stigmatic contact). Instead, longer visits reduced the likelihood of stigma contacts. This suggests that the pollen yield of flowers and pollen deposition on stigmas may not be necessarily linked and positively correlated. In fact, nectarless flowers, which are closely related to buzz pollination, compete with pollinators for pollen, which can be used as a reward for pollinators or for sexual reproduction of the plant (Klinkhamer and de Jong 1993; Westerkamp 1997). These two mutually exclusive destinations of pollen would then help to explain why more pollen removal (longer buzzes) does not necessarily lead to more contact with the stigma. For example, learning to handle flowers in bumblebees increased the efficiency of pollen collection by the bees, but was not associated with pollen deposition on stigmas (Mayberry et al. 2024). However, we did not measure other acoustic characteristics of flower buzzes (amplitude and frequency), among which amplitude has been shown to influence pollen ejection in several plant species (De Luca and Vallejo‐Marín 2013; Tayal and Kariyat 2021; Vallejo‐Marín 2022). A more detailed understanding of the buzz‐pollination community will require a detailed characterization of the biomechanics of vibrations across bee species.

### 4.2 Body Size Emerged as a Key Proxy for Higher Pollination Efficiency

Flower‐buzzing bees, which were exclusively the larger bees, contacted the stigma 10 times more often than non‐buzzing bees and eight times more often than anther‐buzzing bees. Thus, the higher pollination efficiency thus, cannot be explained by the ability to sonicate flowers alone, as both flower‐ and anther‐buzzing bees remove pollen by sonication. Among morphological traits, body size is particularly relevant, as it has been linked to pollination efficiency in several studies (Garibaldi et al. 2015; Solís‐Montero and Vallejo‐Marín 2017; Jauker et al. 2016; Földesi et al. 2020; Mesquita‐Neto et al. 2021). In fact, a visitor is most likely to touch anthers and stigma when its size exceeds the distance between both structures (see Solís‐Montero and Vallejo‐Marín 2017; Mesquita‐Neto et al. 2021; Rego et al. 2024). Contact with both reproductive parts during flower visits is often used as an indicator of bee pollination efficiency (Bernhardt 1995; Vivarelli et al. 2011), since it generally leads to greater pollen deposition on stigmas (Földesi et al. 2020). Furthermore, bee body size is positively correlated with foraging distance, which increases the chances of long‐distance conspecific pollen flow (Greenleaf et al. 2007; Ricketts et al. 2008; Garibaldi et al. 2011). Therefore, bee body size is a key functional trait in the buzz pollination community studied here, as it is strongly positively associated with pollination efficiency, supporting our Hypothesis 1.

### 4.3 Long Handling Times Did Not Improve Pollination Rather Than Reduce It

Despite previous findings suggesting that longer flower handling times are associated with higher pollination success—since prolonged visits can increase the likelihood of stigma contact (Woodcock et al. 2013)—we found the opposite pattern in our buzz‐pollinated plant community. Bees that spent less time handling flowers were more effective pollinators of plants with poricidal anthers than those making longer visits, thereby rejecting our second hypothesis. This result is consistent with Solís‐Montero et al. (2015), who found that in *Solanum rostratum* , a buzz‐pollinated plant, legitimate floral visitors tend to spend less time on flowers than illegitimate ones. The most likely explanation for this unexpected pattern is that flower‐buzzing bees, which extract pollen more efficiently, are able to visit more flowers in the same amount of time (Amorim et al. 2019; Mesquita‐Neto et al. 2021). This behavior increases the likelihood of transferring pollen between conspecific plants, thereby promoting cross‐pollination (Heinrich and Raven 1972). In line with this, flower‐buzzing bees visited an average of 4.5 flowers per minute, compared to just 1.0 and 1.4 flowers per minute for non‐buzzing and anther‐buzzing bees, respectively. These fast‐foraging, large‐bodied bees also visited significantly more flowers per foraging bout than bees from other groups.

Although both flower‐ and anther‐buzzing bees share the sonication pollen extraction mechanism, anther‐buzzing bees behave more like non‐buzzing bees. They reposition themselves on flowers to sonicate different sets of anthers (Mesquita‐Neto et al. 2018), which might intuitively seem to increase the chances of contacting the stigma. However, our data do not support this: anther‐buzzing bees contacted stigmas 1.8 times less frequently than flower‐buzzing bees, and showed no significant difference from non‐buzzing bees. In addition, anther‐buzzing bees visited about three times fewer plants than flower‐buzzing bees—twice the difference observed between anther‐buzzing and non‐buzzing bees (1.5 more plants per minute). Therefore, anther‐buzzing bees were less efficient pollinators, despite their buzzing ability and overall moderate body size. In fact, we found an overall negative relationship between bee body size and the duration of visits at both the flower and plant levels, with smaller bees spending more time on a single flower and within the same plant individual (see Figure 2). In brief, shorter flower and plant handling times, together with large body size and buzzing behavior at the flower level, were important predictors of pollination efficiency in bees visiting plants with poricidal anthers. Consequently, non‐buzzing and single anther‐buzzing bees are likely to be relatively ineffective pollinators, especially for obligate outbreeding species with mass flowering or multi‐flowered inflorescences, where successful pollination depends on efficient pollen transfer between conspecific plant individuals. Their low rates of stigma contact, combined with limited flower visitation and smaller body size, reduce the likelihood of effective cross‐pollination, and highlight the potential for size‐dependent pollen theft in buzz‐pollinated systems.

Our approach, using behavioral and morphological traits of a visiting bee assemblage involved in pollen collection from flowers with poricidal anthers, helps to explain the pollination efficiencies of the different bee species. However, the large number of species involved and the short co‐flowering period exacerbate the difficulties in obtaining more detailed information, such as testing whether these traits change with differences in plant floral traits. This information could help to clarify whether bees manipulating flowers extract more pollen per unit time on plants with numerous low‐pollen flowers than on more time‐consuming visits to richer‐pollen flowers. Unfortunately, we do not have information on the breeding systems of most of the plant species included in this study. This is particularly important to confirm the importance of faster flower‐visiting bees for cross‐pollination and fruit‐set promotion. Obligate xenogamous species would more obviously benefit from bees that visit more flowers and therefore more plants per time. Morphological or molecular analysis of pollen loads in the bee body could be a supporting measure to verify their floral constancy (Bell et al. 2017; Carneiro et al. 2024; Cirtwill et al. 2024; Tourbez et al. 2024). Finally, the measure of stigma contact used as a proxy for pollination efficiency is a simplified measure and can eventually lead to misinterpretation. However, in community studies with a large number of independent observations, stigma contact per visit, together with flower and plant handling times, is the most reliable simplified proxy for pollination efficiency. We acknowledge that this approach focuses solely on female reproductive success and does not account for male fitness components, such as pollen removal or the quantity of pollen carried by bees. Including such metrics, particularly the quantification of pollen loads and the frequency of visits, would provide a more comprehensive evaluation of pollinator effectiveness, particularly in buzz‐pollinated systems where pollen theft may occur. Therefore, we recommend that future studies integrate pollen deposition and pollen removal metrics to capture the full spectrum of pollination services more effectively (also see Vallejo‐Marin and Russell 2023). Furthermore, studies in other buzz‐pollinated communities from different geographical regions and using more reliable measures of pollinator performance (e.g., single visit pollen deposition) would help generalize our findings.

In summary, we found that morphological and behavioral traits of flower‐visiting bees can predict pollination success in a plant community associated with the buzz‐pollination syndrome. Although bees associated with plants with poricidal anthers may have the same pollen extraction behavior, differences in bee traits are joint forces driving the interactions between partners. In contrast to non‐buzzing bees, larger body size and faster handling time of buzzing bees are important predictors of efficient foraging behavior and also of effective pollination of flowers with poricidal anthers.

## Author Contributions

**José Neiva Mesquita‐Neto:** conceptualization (lead), data curation (lead), formal analysis (lead), funding acquisition (equal), investigation (lead), methodology (equal), software (lead), visualization (lead), writing – original draft (lead), writing – review and editing (equal). **Clemens Schlindwein:** conceptualization (supporting), funding acquisition (lead), investigation (supporting), methodology (equal), project administration (lead), supervision (lead), validation (lead), writing – review and editing (lead).

## Conflicts of Interest

The authors declare no conflicts of interest.

## Supporting information


**Table S1:** Intertegular span and total number of individuals collected per bee species (buzzing and non‐buzzing) visiting buzz‐pollinated flowers in Rio Preto State Park.

## Data Availability

The datasets generated during this study are available in the Figshare repository at: https://doi.org/10.6084/m9.figshare.23648916.
