# Supplementary material for: Bee Body Size and Foraging Behavior Predict the Pollination Role of Bees in a Buzz‐Pollinated Plant Community
Source: Ecol Evol. 2025 Sep 8;15(9):e72150. doi: 10.1002/ece3.72150 (PMC12417104; doi:10.1002/ece3.72150)
Supplement: Supplementary file 1 — Table S1: Intertegular span and total number of individuals collected per bee species (buzzing and non‐buzzing) visiting buzz‐pollinated flowers in Rio Preto State Park. [file ECE3-15-e72150-s001.docx]

**Table S1:** Intertegular span and total number of individuals collected per bee species (buzzing and non-buzzing) visiting buzz-pollinated flowers in Rio Preto State Park.

| **Functional group / species** | **Mean intertegular span (± SD)** | **Number of individuals in flowers** |
| --- | --- | --- |
| **Anther buzzing bees** | | |
| *Augochloropsis* sp.1 | 2.92 (**±**1.30) | 6 |
| *Augochloropsis* sp.2 | 2.42 (±0.04) | 2 |
| *Augochloropsis* sp.3 | 3.24 (±0.15) | 3 |
| *Augochloropsis* sp.4 | 3.26 (±0.11) | 13 |
| *Augochloropsis* sp.5 | 3.01 (±0.1) | 2 |
| *Augochloropsis* sp.6 | 3.43 (±0.18) | 7 |
| *Exomalopsis fulvofasciata* | 3.83 | 1 |
| *Melipona quinquefasciata* | 3.73 (±0.31) | 15 |
| *Pseudaugochlora graminea* | 3.34 (±0.15) | 4 |
| *Pseudaugochlora pandora* | 2.50 (±0.20) | 19 |
| *Thygater analis* | 4.41 | 1 |
| **Flower buzzing bees** | | |
| *Bombus brevivillus* | 6.44 (±1.66) | 31 |
| *Bombus morio* | 7.17 (±1.47) | 74 |
| *Bombus pauloensis* | 5.97 (±1.32) | 28 |
| *Centris aenea* | 6.88 | 1 |
| *Centris caxiensis* | 5.89 (±0.12) | 2 |
| *Centris spilopoda* | 5.72 (±0.37) | 9 |
| *Centris fuscata* | 6.49 (±0.39) | 9 |
| *Centris perforator* | 5.90 (±2.06) | 5 |
| *Centris scopipes* | 11.11 | 1 |
| *Centris tarsata* | 4.74 (±0.35) | 14 |
| *Centris trigonoides* | 4.49 (±0.37) | 5 |
| *Centris varia* | 6.18 (±0.21) | 9 |
| *Epicharis bicolor* | 5.71 | 1 |
| *Eufriesea nigrohirta* | 8.91 | 1 |
| *Euglossa melanotricha* | 4.76 (±0.21) | 7 |
| *Eulaema cingulate* | 9.16 (±0.46) | 13 |
| *Eulaema nigrita* | 8.17 (±0.39) | 8 |
| *Oxaea flavescens* | 6.87 (±0.42) | 24 |
| *Ptiloglossa latecalcarata* | 6.18 (±0.21) | 4 |
| *Ptiloglossa matutina* | 6.52 | 1 |
| *Xylocopa abbreviate* | 5.58 (±0.2) | 2 |
| *Xylocopa bimaculata* | 7.29 (±0.3) | 2 |
| *Xylocopa carbonaria* | 7.98 | 1 |
| *Xylocopa cearensis* | 8.29 (±0.26) | 2 |
| *Xylocopa frontalis* | 12.20 (±0.70) | 4 |
| *Xylocopa grisescens* | 11.65 | 1 |
| *Xylocopa hirsutissima* | 9.38 (±1.28) | 22 |
| *Xylocopa nigrocincta* | 9.56 (±0.46) | 10 |
| *Xylocopa subcyanea* | 6.02 | 1 |
| *Xylocopa suspecta* | 6.30 | 1 |
| **Non-buzzing bees** | | |
| *Apis mellifera* | 3.80 | 1 |
| *Frieseomelitta* sp.2 | 1.96 | 1 |
| *Oxytrigona* sp.1 | 1.95 | 1 |
| *Paratrigona* sp.1 | 1.90 | 1 |
| *Partamona* sp.1 | 2.15 (±0.06) | 2 |
| *Partamona* sp.2 | 2.59 | 1 |
| *Tetragonisca angustula* | 1.10 | 1 |
| *Trigona* sp.1 | 2.14 (0.16) | 68 |
